# ASSOCIATION OF COGNITION WITH FALLS RISK MODERATED BY RACE AND SOCIAL DETERMINANTS OF HEALTH

**DOI:** 10.1093/geroni/igae098.4121

**Published:** 2024-12-31

**Authors:** Sheila Black, Olivio Clay, Tyler Bell, Jason Blake, Kyle Kraemer, Barbara Jackson, Heaven Cauble, Teairra Evans

**Affiliations:** The University of Alabama, Tuscaloosa, Alabama, United States; The University of Alabama at Birmingham, Birmingham, Alabama, United States; University of California San Diego, San Diego, California, United States; The University of Alabama at Birmingham, Birmingham, Alabama, United States; Texas Education Agency, Austin, Texas, United States; NORC, Chicago, Illinois, United States; The University of Alabama, Tuscaloosa, Alabama, United States; Louisiana State University, Baton Rouge, Louisiana, United States

## Abstract

This study examined the extent to which social determinants of health and race moderated the effects of cognitive decline on fall risk. Our sample included 681 participants from the control arm of the Advanced Cognitive Training for Independent and Vital Elderly (ACTIVE) study who received five assessments across ten years. Participants in this study received measures to assess their cognitive status and history of falls. The cognitive evaluation included measures to assess processing speed, reasoning ability, and episodic memory. The active database included measures that allowed us to assess social determinants of Health (SDOH). SDOH included economic stability, education, social and community context, access to healthcare, neighborhood and built environment. We found that changes in cognition (e.g., processing speed) were associated with greater fall risk. The effects of cognitive decline were moderated by race and social determinants of health. That is, slower processing speed over time was related to increased fall risk for people with above average educational/ occupational status, access to health care, and social community contexts. Regarding race, cognitive decline over time was related to a lower fall risk in White older adults but higher fall risk in Black/African American older adults. These findings were discussed in terms of racial disparities in SDOH resources and in terms of the relation between life space and SDOH resources.

